# Nucleobase-Based Barbiturates: Their Protective Effect against DNA Damage Induced by Bleomycin-Iron, Antioxidant, and Lymphocyte Transformation Assay

**DOI:** 10.1155/2014/898670

**Published:** 2014-05-08

**Authors:** Bhaveshkumar D. Dhorajiya, Bharatkumar Z. Dholakiya, Ahmed S. Ibrahim, Farid A. Badria

**Affiliations:** ^1^Department of Applied Chemistry, S.V. National Institute of Technology, Ichchhanath, Surat, Gujarat 395007, India; ^2^Department of Biochemistry, Faculty of Pharmacy, Mansoura University, Mansoura 35516, Egypt; ^3^Department of Pharmacognosy, Faculty of Pharmacy, Mansoura University, Mansoura 35516, Egypt

## Abstract

A number of nucleobase-based barbiturates have been synthesized by combination of nucleic acid bases and heterocyclic amines and barbituric acid derivatives through green and efficient multicomponent route and one pot reaction. This approach was accomplished efficiently using aqueous medium to give the corresponding products in high yield. The newly synthesized compounds were characterized by spectral analysis (FT-IR, ^1^H NMR, ^13^C NMR, HMBC, and UV spectroscopy) and elemental analysis. Representative of all synthesized compounds was tested and evaluated for antioxidant, bleomycin-dependent DNA damage, and Lymphocyte Transformation studies. Compounds **TBC** > **TBA** > **TBG** showed highest lymphocyte transformation assay, **TBC** > **TBA** > **BG** showed inhibitory antioxidant activity using ABTS methods, and **TBC** > **BPA** > **BAMT** > **TBA** > **1**, **3** **-TBA** manifested the best protective effect against DNA damage induced by bleomycin.

## 1. Introduction

The area of free radical biology and medicine is developing fast, since the discovery of the involvement of free radicals in oxidative tissue injury and diseases. Free radicals and other reactive oxygen species such as superoxide radical anion, hydroxyl radical, and hydrogen peroxide are constantly generated through many biological processes and may be considered as a measure of biological inefficiency [1]. The human body uses an antioxidant system to neutralize the excessive levels of reactive oxygen species that consists of enzymes such as superoxide dismutases, catalases, and glutathione peroxidases, in addition to numerous nonenzymatic small molecules that are widely distributed in the biological system such as glutathione, *α*-tocopherol, ascorbic acid, *β*-carotene, and selenium [2]. In general, the cell is able to maintain an appropriate balance between oxidants and antioxidants under normal conditions. The imbalance between reactive oxygen species production and the available antioxidant defence leads to a widely accepted phenomenon called oxidative stress [3]. Barbiturates have been in the center of attention of researchers over many years because of the high practical value of these compounds. In the first place, barbiturates are very important class of compounds, for their high reactivity in synthesis, as key starting materials to form various classes of biologically and pharmacologically active candidates. The diverse biologic activity and coverage of a broad chemical space make barbituric acid and thiobarbituric acid derivatives excellent target compounds for organic and medicinal chemists. Owing to their ready availability and various functionalization possibilities, the parent barbituric acid and thiobarbituric acid are convenient starting materials for the preparation of different fused heterocycles and literature survey also describe that 5-substituted derivatives are pharmacologically active compounds [4]. The medicinal importance of pyrimidine derivatives such as barbituric acid and thiobarbituric acid plays vital role among various heterocyclic compounds due to their antineoplastic [5, 6], antiviral [7], antibiotic [8], and anti-inflammatory [9] activity. The pyrimidine ring system is present in various natural compounds such as nucleic acids, vitamins, coenzymes, uric acid, purines, and some marine microorganisms (e.g., sponge) [10]. Cyclic imides are an important class of molecules known for their diverse array of bioactivities. These biological activities and various pharmacological uses of cyclic imides have been attributed to their unique structural features [11]. In view of these facts and in continuation of our interest in the synthesis of a variety of heterocycles of biological importance, we report here an efficient and convenient method for the synthesis of novel nucleobased barbiturates derivatives attached to pyrimidine moiety. The synthesized compounds were evaluated for their antioxidant protection activities against DNA damage induced by bleomycin-iron assay, and this may help to reduce the side effects of chemotherapy for cancer patients.

## 2. Materials and Methods

Chemicals and solvents were obtained from commercial sources and used as received throughout the investigation. The barbituric acid was synthesized by using diethyl malonate and urea using standard procedure. Melting points were determined in open capillaries on a Veego electronic apparatus VMP-D (Veego Instrument Corporation, Mumbai, India) and are uncorrected. IR spectra (4000–400 cm^−1^) of synthesized compounds were recorded on a Perkin Elmer-Spectrum RX-IFTIR spectrophotometer using KBr pellets. Thin layer chromatography was performed on object glass slides (2 × 7.5 cm) coated with silica gel-G and spots were visualized under UV irradiation. ^1^H NMR and ^13^C NMR spectra were recorded on an Avance-II (Bruker) model using DMSO as a solvent and TMS as internal standard with ^1^H resonant frequency of 400 MHz and ^13^C resonant frequency of 100 MHz. The ^1^H NMR and ^13^C NMR chemical shifts were reported as parts per million (ppm) downfield from TMS (Me_4_Si). The splitting patterns are designated as follows: s, singlet; br s, broad singlet; d, doublet; t, triplet; q, quartet; m, multiplet. U.V. spectra were recorded on Maya pro 2000 (Ocean Optics, USA) using DMSO as a solvent with 10^−5 ^M solution.

### 2.1. Synthesis of Barbituric Acid

**Scheme 1:**
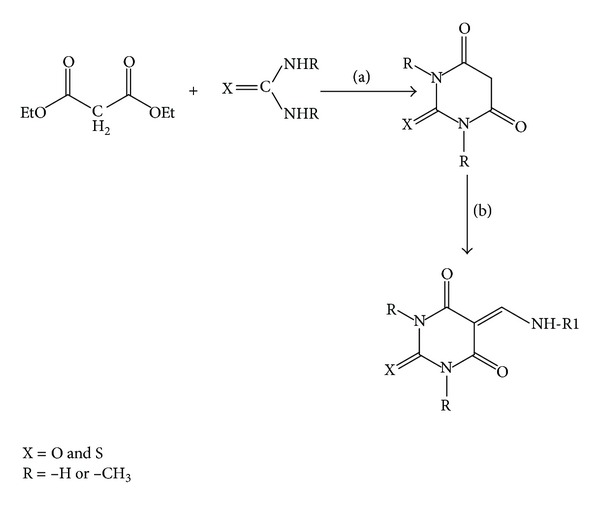
Synthesis of DNA base and heterocyclic primary amines-based barbiturates. Reagents and conditions: (a) NaOMe/MeOH-refluxed (b) HCO_2_H, water, reflux, where R1 = adenine, guanine, cytosine, and primary heterocyclic amine.

As shown in Scheme 1. To a solution of diethylmalonate (20 g, 118.9 mmol) and urea, thiourea and its methyl substituted analogues were (7.5 g, 98.5 mmol) in methanol; anhydrous sodium methoxide was added and refluxed at 65°C for 8 h. A white solid separates. Then, in the above reaction mixture, 125 mL of hot (50°C) water was added and hydrochloric acid was used to make the solution acidic. After the completion of the reaction, the resulting clear solution was filtered and cooled in an ice bath overnight. The white product formed was filtered, washed with 50 mL of cold water, dried, and recrystallized from acetone to afford compound as a white powder [12].

#### 2.1.1. Synthesis of DNA-Based Barbiturates

As shown in Scheme 1, a solution of the corresponding DNA-bases (e.g., adenine 1 mmol) and formic acid (4 mmol) in distilled water, as a green solvent, was allowed to reflux at 60°C over a period of 2-3 h. Then, it was cooled down until the reaction mixture becomes a clear solution. The completion of reaction was confirmed by TLC. In situ barbiturates were added again and refluxed further for 3-4 h. As the reaction proceeds, the solid products were separated out in the form of suspension and the precipitated DNA-based barbiturates were separated by filtration, washed with water three times followed by n-hexane, and then dried in a desiccators [12].

### 2.2. Characterization Data of Synthesized Compounds

#### 2.2.1. 5-[(9H-Purin-6-ylamino)-methylene]-pyrimidine-2,4,6-trione (BA)

Yellow powder, yield 77%; m.p. >250°C; ^1^H NMR (400 MHz, DMSO), 4.02 *δ* ppm (1H, dd, exocyclic NH of purine ring, *j* = 16.80 Hz), 6.88 *δ* ppm (2H, s, endocyclic-NH of purine ring), 8.08 *δ* ppm (1H, dd, exocyclic CH of pyrimidine ring, *j* = 25.36 Hz), 8.14 *δ* ppm (1H, dd, NH of purine ring), 11.21 *δ* ppm (1H, s, NH of pyrimidine ring), 11.22 *δ* ppm (1H, s, NH of pyrimidine ring). ^13^C NMR (400 MHz, DMSO), 77 (C-5), 114.32 (C-9), 114.67 (C-8), 122.95 (C-13), 127.12 (C-12, C-14), 129.27 (C-11, C-15), 132 (C-10), 155 (C-7), 163.15 (C-4, C-6), 168.05 (C-2) *δ* ppm. FTIR (KBr) *υ*_max⁡_ cm^−1^: 1212 (m, -O-C stretching), 1631 (s, =C-NH aliphatic amine), 1693 (s, C=O), 2826 (exocyclic CH), 3073 (m, CH-NH stretching). *λ* max: 303.22 nm; (€: 1.10 × 10^5^ L mol^−1^ cm^−1^); M.W.273.21, ESIMS: *m*/*z* 274.25 (M + 1); Anal. Calcd. For C_10_H_7_N_7_O_3_ (%): C 43.96, H 2.58, N 35.89. Found (%): C 43.94, H 2.57, N 35.91.

#### 2.2.2. 5-[(9H-Purin-6-ylamino)-methylene]-2-thioxo-dihydro-pyrimidine-4,6-dione (TBA)

Yellow powder, yield 58%; m.p. > 250°C; ^1^H NMR (400 MHz, DMSO), 4.04 *δ* ppm (1H, dd, exocyclic NH of purine ring, *j* = 15.85 Hz), 6.92 *δ* ppm (2H, s, -CH of purine ring), 8.12 *δ* ppm (1H, dd, exocyclic CH of pyrimidine ring, *j* = 24.60 Hz), 8.20 *δ* ppm (1H, dd, NH of purine ring), 11.18 *δ* ppm (1H, s, NH of pyrimidine ring), 11.21 *δ* ppm (1H, s, NH of pyrimidine ring). ^13^C NMR (400 MHz, DMSO), 76 (C-5), 115.10 (C-9), 115.40 (C-8), 122.82 (C-13, C-14), 126.72 (C-12, C-15), 129.67 (C-11), 131.89 (C-10) 155.15 (C-7), 165.15 (C-4, C-6), 168.25 (C-2) *δ* ppm. FTIR (KBr) *υ*_max⁡_ cm^−1^: 1216 (m, -O-C stretching), 1628 (s, =C-NH aliphatic amine), 1698 (s, C=O), 2808 (exocyclic CH), 3075 (m, CH-NH stretching). *λ* max: 299 nm; (€: 1.03 × 10^5^ L mol^−1^ cm^−1^); M.W.289.27, ESIMS: *m*/*z* 289.05 (M); Anal. Calcd. For C_10_H_7_N_7_O_2_S (%): C 41.52, H 2.44, N 33.89. Found (%): C 42.48, H 2.48, N 33.90.

#### 2.2.3. 1,3-Dimethyl-5-[(9H-purin-6-ylamino)-methylene]-pyrimidine-2,4,6-trione (1,3 BA)

Yellow powder, yield 82%; m.p. >250°C; ^1^H NMR (400 MHz, DMSO), 2.72 (6H, s, two CH_3_ group of pyrimidine ring), 5.67 *δ* ppm (1H, dd, exocyclic NH of purine ring, *j* = 14.08 Hz), 6.85 *δ* ppm (2H, s, -CH of purine ring), 8.09 *δ* ppm (1H, dd, exocyclic CH of pyrimidine ring, *j* = 24.94 Hz), 8.16 *δ* ppm (1H, dd, NH of purine ring). ^13^C NMR (400 MHz, DMSO), 78 (C-5), 113.10 (C-9), 113.38 (C-8), 121.39 (C-13), 126.56 (C-12, C-14), 129.60 (C-11, C-15), 133.29 (C-10), 157.55 (C-7), 163.15 (C-4, C-6), 168.05 (C-2) *δ* ppm. FTIR (KBr) *υ*_max⁡_ cm^−1^: 1208 (m, -O-C stretching), 1628 (s, =C-NH aliphatic amine), 1695 (s, C=O), 2805 (m, -N-CH_3_ stretching), 2816 (exocyclic CH), 3070 (m, CH-NH stretching). *λ* max: 303.22 nm; (€: 1.00 × 10^5^ L mol^−1^ cm^−1^); M.W. 301.26, ESIMS: *m*/*z* 302.25 (M + 2); Anal. Calcd. For C_12_H_11_N_7_O_3_ (%): C 47.84, H 3.68, N 32.55. Found (%): C 47.81, H 3.70, N 32.56.

#### 2.2.4. 1,3-Dimethyl-5-[(9H-purin-6-ylamino)-methylene]-2-thioxo-dihydro-pyrimidine-4,6-dione (1,3-TBA)

Dark orange powder, yield 62%; m.p. >250°C; ^1^H NMR (400 MHz, DMSO), 2.78 (6H, s, two CH_3_ group of pyrimidine ring), 4.03 *δ* ppm (1H, dd, exocyclic NH of purine ring, *j* = 15.28 Hz), 6.90 *δ* ppm (2H, s, -CH of purine ring), 8.14 *δ* ppm (1H, dd, exocyclic CH of pyrimidine ring, *j* = 23.34 Hz), 8.18 *δ* ppm (1H, dd, NH of purine ring). ^13^C NMR (400 MHz, DMSO), 58.08 (C-18), 78.85 (C-5), 113.30 (C-9), 113.47 (C-8), 122.95 (C-11, C-15), 129.27 (C-12, C-14), 131.17 (C-10, C-13), 157 (C-7), 162.66 (C-4, C-6), 172.05 (C-2) *δ* ppm. *λ* max: 288.21 nm; (€: 0.90 × 10^5^ L mol^−1^ cm^−1^); M.W. 317.33, FTIR (KBr) *υ*_max⁡_ cm^−1^: 1220 (m, -O-C stretching), 1635 (s, =C-NH aliphatic amine), 1702 (s, C=O), 2796 (m, -N-CH_3_ stretching), 2812 (exocyclic CH), 3081 (m, CH-NH stretching). M.W. 317.07, ESIMS: *m*/*z* 309.06 (M + 2); Anal. Calcd. C_12_H_11_N_7_O_2_S (%): C 45.42, H 3.49, N 30.90. Found (%): C 42.40, H 3.52, N 30.92.

#### 2.2.5. 5-[(2-Hydroxy-9H-purin-6-ylamino)-methylene]-pyrimidine-2,4,6-trione (BG)

Yellow powder, yield 74%; m.p. 250°C; ^1^H NMR (400 MHz, DMSO), 4.04 *δ* ppm (1H, dd, exocyclic NH of purine ring, *j* = 15.68 Hz), 6.97 *δ* ppm (1H, s, -CH of purine ring), 8.06 *δ* ppm (1H, dd, exocyclic CH of pyrimidine ring, *j* = 24.76 Hz), 8.15 *δ* ppm (1H, dd, NH of purine ring), 11.15 *δ* ppm (1H, s, NH of pyrimidine ring), 11.27 *δ* ppm (1H, s, NH of pyrimidine ring), 12.30 *δ* ppm (1H, s, OH of purine ring). ^13^C NMR (400 MHz, DMSO), 58.08 (C-18, C-17) 75.35 (C-5), 114.56 (C-9), 114.87 (C-8), 122.95 (C-11, C-15), 129.27 (C-12, C-14), 131.17 (C-10, C-13), 158 (C-7), 162.66 (C-4, C-5), 167.05 (C-2) *δ* ppm. FTIR (KBr) *υ*_max⁡_ cm^−1^: 1221 (m, -O-C stretching), 1629 (s, =C-NH aliphatic amine), 1691 (s, C=O), 3070 (m, CH-NH stretching) 3345 (C-OH). *λ* max: 276 nm; (€: 0.95 × 10^5^ L mol^−1^ cm^−1^); M.W. 289.21, ESIMS: *m*/*z* 289.20 (M); Anal. Calcd. For C_10_H_7_N_7_O_4_ (%): C 41.53, H 2.44, N 33.90. Found (%): C 42.49, H 2.46, N 33.89.

#### 2.2.6. 5-[(2-Hydroxy-9H-purin-6-ylamino)-methylene]-2-thioxo-dihydro-pyrimidine-4,6-dione (TBG)

Off-white, yield 84%; m.p. >250°C; ^1^H NMR (400 MHz, DMSO), 4.09 *δ* ppm (1H, dd, exocyclic NH of purine ring, *j* = 14.98 Hz), 6.93 *δ* ppm (2H, s, -CH of purine ring), 8.03 *δ* ppm (1H, dd, exocyclic CH of pyrimidine ring, *j* = 23.26 Hz), 10.97 *δ* ppm (1H, dd, NH of purine ring), 11.17 *δ* ppm (1H, s, NH of pyrimidine ring), 11.26 *δ* ppm (1H, s, NH of pyrimidine ring), 12.36 *δ* ppm (1H, s, OH of purine ring). ^13^C NMR (400 MHz, DMSO), 74.53 (C-5), 113.26 (C-9), 113.59 (C-8), 122.92 (C-13, C-14), 126.51 (C-12, C-15), 129.39 (C-11), 131.78 (C-10) 157.23 (C-7), 162.66 (C-4, C-6), 168.62 (C-2) *δ* ppm. FTIR (KBr) *υ*_max⁡_ cm^−1^: 1215 (m, -O-C stretching), 1362 (s, C-NH, aromatic amine), 1632 (s, =C-NH aliphatic amine), 1698 (s, C=O), 2821 (exocyclic CH), 3076 (m, CH-NH stretching), 3342 (b, C-OH). *λ* max: 291.03 nm; (€: 0.95 × 10^5^ L mol^−1^ cm^−1^); M.W. 305.27, ESIMS: *m*/*z* 306.29 (M + 1); Anal. Calcd. C_10_H_7_N_7_O_3_S (%): C 39.34, H 2.31, N 32.12. Found (%): C 39.31, H 2.35, N 32.09.

#### 2.2.7. 5-(((2-Hydroxypyrimidin-4-yl)amino)methylene)pyrimidine-2,4,6-(1H,3H,5H)-trione (BC)

Yellow powder, yield 82%; m.p. >250°C; ^1^H NMR (400 MHz, DMSO), 4.06 *δ* ppm (1H, dd, exocyclic NH of purine ring, *j* = 15.68 Hz), 6.98 *δ* ppm (2H, s, -CH of cytosine ring), 8.11 *δ* ppm (1H, dd, exocyclic CH of pyrimidine ring, *j* = 23.60 Hz), 8.16 *δ* ppm (1H, dd, NH of cytosine ring), 11.18 *δ* ppm (1H, s, NH of pyrimidine ring), 11.26 *δ* ppm (1H, s, NH of pyrimidine ring). ^13^C NMR (400 MHz, DMSO), 78.53 (C-7), 113.26 (C-9), 113.68 (C-8), 121.29 (C-13), 126.43 (C-12, C-14), 129.58 (C-11, C-15), 133.18 (C-10) 157.53 (C-7), 161.35 (C-4, C-6), 169.34 (C-2) *δ* ppm. FTIR (KBr) *υ*_max⁡_ cm^−1^: 1209 (m, -O-C stretching), 1354 (s, C-NH, aromatic amine), 1632 (s, =C-NH aliphatic amine), 1694 (s, C=O aromatic and *α*, *β*-unsaturated ketone), 3072 (m, CH-NH stretching). *λ* max: 286.34 nm; (€: 1.14 × 10^5^ L mol^−1^ cm^−1^); M.W. 249.18, ESIMS: *m*/*z* 251.14 (M + 2); Anal. Calcd. for C_9_H_7_N_5_O_4_ (%): C 43.38, H 2.83, N 28.11. Found (%): C 43.40, H 2.79, N 28.12.

#### 2.2.8. 5-((2-Hydroxypyrimidine-4-ylamino)methylene)-dihydro-2-thioxopyrimidine-4,6(1H,5H)-dione (TBC)

Dark orange powder, yield 75%; m.p. >250°C; ^1^H NMR (400 MHz, DMSO), 4.09 *δ* ppm (1H, dd, exocyclic NH of purine ring, *j* = 13.8 Hz), 6.95 *δ* ppm (2H, s, -CH of cytosine ring), 8.07 *δ* ppm (1H, dd, exocyclic CH of pyrimidine ring, *j* = 23.66 Hz), 8.18 *δ* ppm (1H, dd, NH of cytosine ring), 11.12 *δ* ppm (1H, s, NH of pyrimidine ring), 11.19 *δ* ppm (1H, s, NH of pyrimidine ring). ^13^C NMR (400 MHz, DMSO), 77 (C-5), 113.45 (C-9), 113.76 (C-8), 121.78 (C-13), 126.34 (C-12, C-14), 129.87 (C-11, C-15), 132.22 (C-10), 158.25 (C-7), 166.15 (C-4, C-6), 169.26 (C-2) *δ* ppm. FTIR (KBr) *υ*_max⁡_ cm^−1^: 1213 (m, -O-C stretching), 1626 (s, =C-NH aliphatic amine), 1699 (s, C=O), 2809 (exocyclic CH), 3078 (m, CH-NH stretching). *λ* max: 284.46 nm; ESIMS: *m*/*z* 284.45 (M); (€: 1.07 × 10^5^ L mol^−1^ cm^−1^); M.W. 265.25, Anal. Calcd. C_9_H_7_N_5_O_3_S (%): C 40.75, H 2.66, N 26.40. Found (%): C 40.71, H 2.69, N 26.42.

#### 2.2.9. 5-((1,6-Dihydro-2-hydroxypyrimidine-4-ylamino)-methylene)-1,3-dimethylpyrimidine-2,4,6(1H,3H,5H)-trione (1,3-BC)

Yellow powder, yield 65%; m.p. >250°C; ^1^H NMR (400 MHz, DMSO), 2.73 (6H, s, two CH_3_ group of pyrimidine ring), 4.07 *δ* ppm (1H, dd, exocyclic NH of purine ring, *j* = 14.52 Hz), 6.92 *δ* ppm (2H, s, -CH of cytosine ring), 8.13 *δ* ppm (1H, dd, exocyclic CH of pyrimidine ring, *j* = 23.62 Hz), 8.15 *δ* ppm (1H, dd, NH of cytosine ring). ^13^C NMR (400 MHz, DMSO), 26.6 (N-CH_3_), 35.9 (C-11), 79.5 (C-5), 96.5 (C-12), 140.9 (C-8), 146.1 (C-7), 162.4 (C-4, C-6), 169.54 (C-10), 172.6 (C-2) *δ* ppm. FTIR (KBr) *υ*_max⁡_ cm^−1^: 1217 (m, -O-C stretching), 1634 (s, =C-NH aliphatic amine), 1708 (s, C=O), 2809 (m, -N-CH_3_ stretching), 2824 (exocyclic CH), 3082 (m, CH-NH stretching). *λ* max: 292.44 nm; (€: 1.05 × 10^5^ L mol^−1^ cm^−1^); M.W. 277.24, ESIMS: *m*/*z* 278.22 (M + 1); Anal. Calcd. C_11_H_13_N_5_O_4_ (%): C 47.31, H 4.69, N 25.08. Found (%): C 47.29, H 4.74, N 25.09.

#### 2.2.10. 5-((1,6-Dihydro-2-hydroxypyrimidin-4-ylamino)methylene)-dihydro-1,3-dimethyl-2-thioxopyrimidine-4,6(1H,5H)-dione (1,3-TBC)

Orange powder, yield 65%; m.p. >250°C; ^1^H NMR (400 MHz, DMSO), 2.78 (6H, s, two CH_3_ group of pyrimidine ring), 4.04 *δ* ppm (1H, dd, exocyclic NH of purine ring, *j* = 16.28 Hz), 6.89 *δ* ppm (2H, s, -CH of cytosine ring), 8.17 *δ* ppm (1H, dd, exocyclic CH of pyrimidine ring, *j* = 24.62 Hz), 8.20 *δ* ppm (1H, dd, NH of cytosine ring). ^13^C NMR (400 MHz, DMSO), 34.6 (N-CH_3_), 39.7 (C-11), 78.8 (C-5), 97.5 (C-12), 139.6 (C-8), 144.5 (C-7), 161.7 (C-4, C-6), 169.54 (C-8), 173.6 (C-2) *δ* ppm. FTIR (KBr) *υ*_max⁡_ cm^−1^: 1212 (m, -O-C stretching), 1628 (s, =C-NH aliphatic amine), 1691 (s, C=O), 2797 (m, -N-CH_3_ stretching), 2813 (exocyclic CH), 3068 (m, CH-NH stretching). *λ* max: 281.17 nm; (€: 0.95 × 10^5^ L mol^−1^ cm^−1^); M.W. 293.30, ESIMS: *m*/*z* 295.27 (M + 2); Anal. Calcd. C_11_H_13_N_5_O_3_S (%): C 44.74, H 4.44, N 23.71, Found (%): C 44.71, H 4.46, N 23.70.

#### 2.2.11. 5-((Pyridin-2-ylamino)methylene)pyrimidine-2,4,6(1H,3H,5H)-trione (2-BAP)

Yellow powder, yield 65%; m.p. >250°C; ^1^H NMR (400 MHz, DMSO), 4.05 *δ* ppm (1H, dd, exocyclic NH of pyridine ring, *j* = 16.38 Hz), 6.59–8.09 *δ* ppm (4H, m, -CH of Pyridine ring), 8.23 *δ* ppm (1H, dd, exocyclic CH of pyrimidine ring, *j* = 23.68 Hz), 10.89 *δ* ppm (1H, s, NH of pyrimidine ring), 11.21 *δ* ppm (1H, s, NH of pyrimidine ring). ^13^C NMR (400 MHz, DMSO), 80.2 (C-5), 108.5 (C-13), 113.7 (C-11), 138.2 (C-12), 147.8, (C-10), 151.3 (C-2), 154.4 (C-7), 164.7 (C-4, C-6) *δ* ppm. FT-IR (KBr) *υ*_max⁡_ cm^−1^: 1218 (m, -O-C stretching), 1622 (=C-NH aliphatic amine), 1697 (C=O), 2815 (exocyclic CH), 3078 (CH-NH stretching). *λ* max: 288.17 nm; (€: 0.95 × 10^5^ L mol^−1^ cm^−1^); M.W. 232.20, ESIMS: *m*/*z* 232.18 (M); Anal. Calcd. C_10_H_8_N_4_O_3_ (%): C 51.73, H 3.47, N 24.13, Found (%): C 51.74, H 3.45, N 24.15.

#### 2.2.12. 5-((5-Methylthiazol-2-ylamino)methylene)pyrimidine-2,4,6(1H,3H,5H)-trione (BAMT)

Orange powder, yield 65%; m.p. >250°C; ^1^H NMR (400 MHz, DMSO), 2.39 (3H, s, -CH_3_ group of thiazol ring), 4.06 *δ* ppm (1H, dd, exocyclic NH of thiazol ring, *j* = 15.38 Hz), 7.17 *δ* ppm (1H, s, -CH of thiazol ring), 8.17 *δ* ppm (1H, dd, exocyclic CH of pyrimidine ring, *j* = 24.36 Hz), 10.96 *δ* ppm (1H, s, NH of pyrimidine ring), 11.26 *δ* ppm (1H, s, NH of pyrimidine ring). ^13^C NMR (400 MHz, DMSO), 17.6 (-CH_3_), 78.8 (C-5), 120.7 (C-11), 134.9 (C-12), 150.4 (C-2), 161.7 (C-9), 165.3 (C-4, C-6), 167.5 (C-7) *δ* ppm. FT-IR (KBr) *υ*_max⁡_ cm^−1^: 1215 (m, -O-C stretching), 1256 (C-S), 1625 (=C-NH aliphatic amine), 1693 (C=O), 2236 (C-N of thiazol ring), 2794 (-CH_3_ stretching), 2813 (exocyclic CH), 3073 (CH-NH stretching). *λ* max: 281.17 nm; (€: 0.95 × 10^5^ L mol^−1^ cm^−1^); M.W. 252.25, ESIMS: *m*/*z* 253.05 (M + 1); Anal. Calcd. C_9_H_8_N_4_O_3_S (%): C 42.85, H 3.20, N 22.21, Found (%): C 42.82, H 3.21, N 22.23.

### 2.3. Pharmacology

#### 2.3.1. Biological Evaluation of the Isolated Compounds

DNA (Calf Thymus type 1), bleomycin sulfate, butylated hydroxyanisole, thiobarbituric acid (TBA), ethylenediaminetetraacetic acid (EDTA), and ascorbic acid were obtained from Sigma. 2,2′-azo-bis-(2-amidinopropane) dihydrochloride and ABTS were purchased from Wako Co., USA.

#### 2.3.2. Antioxidant Screening Assay (ABTS Method)

2,2′-azino-bis-3-ethylbenzthiazol-ine-6-sulfonic acid (ABTS) was purchased from Wako Co., USA. L-ascorbic acid was obtained from Sigma and all other chemicals were of the highest quality available. For each of the investigated compounds (2 mL), ABTS solution (60 *μ*M) was added to 3 mL MnO_2_ solution (25 mg/mL), all prepared in (5 mL) aqueous phosphate buffer solution (pH 7, 0.1 M). The mixture was shaken, centrifuged, and filtered and the absorbance of the resulting green-blue solution (ABTS radical solution) at 734 nm was adjusted to approx. 0.5. Then, 50 *μ*L of (2 mM) solution of the tested compound in spectroscopic grade MeOH/phosphate buffer (1 : 1) was added. The absorbance was measured and the reduction in color intensity was expressed as inhibition percentage. L-ascorbic acid was used as standard antioxidant (positive control). Blank sample was run without ABTS using MeOH/phosphate buffer (1 : 1) instead of tested compounds. Negative control was run with ABTS and MeOH/phosphate buffer (1 : 1) only. The absorbance *A*(test( was measured and the reduction in color intensity was expressed as % inhibition. The inhibition for each compound was calculated from the following equation:

(1) $$\begin{array}{c} \%\;\;\text{inhibition}=\left[\frac{\left\{A\left(\text{control}\right)-A\left(\text{test}\right)\right\}}{A\left(\text{control}\right)}\right]\times 100. \end{array}$$

Ascorbic acid (vitamin C) was used as standard antioxidant (positive control). Blank sample was run without ABTS using methanol/phosphate buffer (1 : 1) instead of sample. Negative control sample was run with methanol/phosphate buffer (1 : 1) instead of tested compound [13, 14].

#### 2.3.3. Antioxidant Activity Screening Assay 2,2′-Azino-bis-3ethylbenzthiazoline-6-sulfonic Acid Method

For each of the investigated compounds, 2 mL of ABTS solution (60 *μ*M) was added to 3 mL MnO_2_ solution (25 mg/mL), all prepared in 5 mL aqueous phosphate buffer solution (pH, 7; 0.1 M). The mixture was shaken, centrifuged, and filtered, and the absorbance of the resulting green-blue solution (ABTS radical solution) at *λ* 734 nm was adjusted to approximately ca. 0.5. Then, 50 *μ*L of (2 mM) solution of the tested compound in spectroscopic grade MeOH/phosphate buffer (1 : 1) was added. The absorbance was measured and the reduction in color intensity was expressed as inhibition percentage. L-ascorbic acid was used as standard antioxidant (positive control). Blank sample was run without ABTS and using MeOH/phosphate buffer (1 : 1) instead of tested compounds [15, 16].

#### 2.3.4. Bleomycin-Dependent DNA Damage Assay

The reaction mixture contained DNA (0.5 mg/mL), bleomycin sulfate (0.05 mg/mL), MgCl_2_ (5 mM), FeCl_3_ (50 mM), and samples to be tested in a conc. of 0.1 mg/mL. L-ascorbic acid was used as positive control. The mixture was incubated at 37°C for 1 hour. The reaction was terminated by addition of 0.05 mL EDTA (0.1 M). The color was developed by adding 0.5 mL TBA (1% w/v) and 0.5 mL HCl (25% v/v), followed by heating at 80°C for 10 minutes. After centrifugation, the extent of DNA damage was measured by increase in absorbance at 532 nm [16].

#### 2.3.5. Lymphocyte Transformation Assay

The viable lymphocytes were adjusted to a concentration of 2 × 10^6^ cells/mL in RPMI-1640 medium supplemented with 600 *μ*L penicillin, 0.1 mL streptomycin, 1% glutamine, 25% HEPES (N-2 hydroxyethylpiperazine-N-D2-ethanesulfonic acid)-buffer, and 20% fetal calf serum (FCS). The lymphocytes were plated into 96-well tissue culture plates (or Eppendorf tubes). 100 *μ*L of the volatile oil solution in DMF (100 *μ*L/mL) and 20 *μ*g of the mitogen (PHA) were added to each well. Cell cultures were incubated at 37°C in 5% CO_2_ atmosphere for 72 h, during which the mitogen produces its maximal effect on DNA synthesis. After culture, 237 cell films were stained by Giemsa stain and average count of percentage of transformed (proliferated) blasts was determined. Aqueous* Echinacea purpurea *extract (Immulon) and levamisole (Ketrax) were used as positive control (standard immunostimulant), 100 *μ*g/mL of each drug in DMSO [17].

## 3. Result and Discussion

### 3.1. Chemistry

The synthetic strategy adopted to obtain the target compounds is depicted in Scheme 1. Various nucleic acid bases such as adenine, guanine, cytosine, and heterocyclic amines were refluxed with formic acid in aqueous medium at 60°C to yield formamide derivatives of corresponding DNA bases. Thereafter, in situ barbituric acid and its analogs were refluxed giving the corresponding DNA based barbiturates [18].

The important infrared spectral bands and their tentative assignments for DNA-based barbiturates were recorded as KBr disks and are presented. The IR spectra of synthesized compounds revealed characteristic bands between 2808 cm^−1^ and 2826 cm^−1^ confirming the presence of exocyclic  CH groups. IR spectrum of the compounds showed characteristic bands between 1691 cm^−1^ and 1708 cm^−1^ confirming the presence of C=O groups.

^1^H NMR spectra revealed signals at 3.50 *δ* ppm for DMSO solvent, between 4.2 and 4.09 *δ* ppm for NH of the DNA base, between 8.03 and 8.17 *δ* ppm for exocyclic CH group, and between 8.09 and 11.27 *δ* ppm for NH of pyrimidine ring. From ^13^C NMR spectra, exocyclic CH signal was observed between 144.5 and 158.25 *δ* ppm. The UV absorption spectra were made using DMSO as a solvent in concentrations (10^−5 ^M). All synthesized new DNA-based barbiturates derivatives showed the strong absorption bands (*λ* max) in the range 275–305 nm owing to the *π*-π* and *n*-π* transitions as well as presence of chromophoric exocyclic CH of pyrimidine ring in their UV spectra [19].

## 4. Pharmacology

A variety of DNA base and heterocyclic primary amines-based barbiturates were tested for antioxidant activity as reflected in the ability to control of ABTS assays. The pro-oxidant activities of the aforementioned derivatives were assessed by their effects on bleomycin-induced DNA damage. The DNA base and heterocyclic primary amines-based barbiturates manifested potent antioxidative activity in the ABTS assay.

All compounds have been tested to bleomycin-dependent DNA damage. The results indicated that they may have some protective activity to DNA by certain mechanism. A series of compounds (**TBC**,** TBA**,** BG**,** 1**,** 3-BA**, and** BAMT**) exhibited a high antioxidant activity. On the other hand, all compounds show potent protective effect on the DNA from the induced damage by bleomycin with reference to ascorbic acid and butylated hydroxyanisole (Table 1).

Lymphocyte transformation assay involves study of a specific immune response at 50 *μ*M. The assay investigates the mitogenic effect of synthesized DNA base and heterocyclic primary amines-based barbiturates on T-lymphocytes. The positive control was* Echinacea purpurea* (Ech) extract at concentration 50 µM giving 74% of lymphocyte transformation. Data obtained from Table 1 indicated compound TBC shows that 68% lymphocyte transformed at concentration similar to standard while 35–55% lymphocyte transformed by synthesized barbiturates** TBA**,** TBG, 1**,**3-BA**,** 1**,**3-TBA**,** BG, and BPA**.

Barbiturate is a versatile moiety that exhibits a wide variety of biological activities. Barbiturate moiety acts as “hydrogen binding domain” and “electron donor system.” It also acts as a constrained pharmacophore. Many drugs containing barbituric acid nucleus are available on the market such as allobarbital, alphenal, thiopental, and cyclobarbital.

Thioxodihydropyrimidine can act as the bioisosteric replacement of the pyrimidine moiety. Members of this ring system have found their way into such diverse application as pharmaceuticals, oxidation inhibitors, cyanine dyes, and metal complexing agents. Barbiturate derivatives have antimicrobial, anti-inflammatory, anticancer, anticonvulsant, antidepressant, antioxidant, radio protective, and antileishmanial activities. From the obtained results (Table 1), it can be concluded that pyrimidine moiety is essential for the protection activity against DNA damage induced by bleomycin-iron complex, and pyrimidine ring is also required for the activity and an unsubstituted phenyl ring exhibited better activity than those substituted derivatives. This good activity of thioxodihydropyrimidine derivatives shows the hypothesis of a direct link between thiol function and an aromatic ring to be a good one. The thiol catches the radical, and afterwards, the aromatic ring permits the trapping of this radical. Moreover, thiobarbituric acid derivative shows a better activity than barbituric acid.

## 5. Conclusion 

In conclusion, we have designed hybrid molecules on the basis of the biological significance of nucleobase and barbituric acid and evaluated their anticancer activities. All of the new synthesized compounds were tested for their protection activity against DNA damage induced by bleomycin-iron complex. Compounds** TBC** and** 2-BAP **showed the highest protection activity against DNA damage. On the other hand, compounds** BA** and** 1**,**3-TBC** showed a very low activity, whereas the rest of the tested compounds exhibited well to moderate activity. It can be concluded that barbituric acid moiety is essential for the protection activity against DNA damage induced by bleomycin-iron complex, and thiobarbituric acid ring is also required for the activity. On the other hand, unsubstituted barbituric acid or thiobarbituric acid exhibited better activity than those substituted derivatives, that is, 1,3-dimethyl substituted derivatives. Out of a set of 12 molecules, four compounds** TBC**,** TBA**,** BG,** and** TBG** exhibit significant antioxidant, bleomycin-dependent DNA damage, and lymphocyte transformation assay activities and could be used as leads for further investigations.

## Figures and Tables

**Table 1:** Biological activities of compounds towards lymphocyte transformation, ABTS (free radical scavenging), and bleomycin-dependent DNA damage activities.

Compound code	Lymphocyte transformation assay at 50 *μ*m^a,b,g^	ABTS (percentage of scavenging inhibition)^c,d,e,f,g^	Bleomycin-dependent DNA damage assay^g,h^ absorbance (*λ* max-532)
Negative control	—	—	—
Butylated hydroxyanisole	—	—	0.0026
Vitamin C (ascorbic acid)	—	—	0.0038 ± 0.01
BA	19	34.3 ± 0.10	0.210 ± 1.12
TBA	53	60.5 ± 0.00	0.014 ± 0.15
1,3-BA	35	41.7 ± 1.15	0.015 ± 0.25
1,3-TBA	35	25.8 ± 1.30	0.014 ± 1.31
BG	37	49.5 ± 0.04	0.017 ± 0.03
TBG	45	33.2 ± 0.70	0.016 ± 0.27
BC	25	31.1 ± 2.38	0.070 ± 0.20
TBC	68	70.8 ± 0.10	0.011 ± 0.04
1,3-BC	20	35.4 ± 0.24	0.017 ± 0.05
1,3-TBC	10	13.3 ± 0.14	0.210 ± 1.18
2-BAP	35	30.7 ± 1.12	0.011 ± 0.27
BAMT	32	40.8 ± 0.10	0.013 ± 0.05

^a^Concentration showing 50% lymphocyte transformation. ^b^The positive control was *Echinacea purpurea* (Ech) extract at concentration 50 giving 74% lymphocyte transformation. ^c^ABTS + scavenging activity (%) = [(Ac − As)/Ac] × 100, where Ac is the absorbance value of the control and As is the absorbance value of the added samples test solution. ^d^The concentration of the pure compounds was 2 mM. ^e^The concentration showing 50% inhibition is expressed in mM. ^f^The positive control was vitamin C and showed 80.0 ± 1.04%. ^g^Values are means of 3 replicates ± SD and showed significant difference at *P* < 0.05 by Student's *t-*test. ^h^The positive control was vitamin C (0.24 mM) and showed absorbance 0.0038 ± 0.01 at the same concentration of the tested compounds.
